# Glycerolized Reticular Dermis as a New Human Acellular Dermal Matrix: An Exploratory Study

**DOI:** 10.1371/journal.pone.0149124

**Published:** 2016-02-26

**Authors:** Pietro Maria Ferrando, Davide Balmativola, Irene Cambieri, Maria Stella Scalzo, Massimiliano Bergallo, Laura Annaratone, Stefania Casarin, Mara Fumagalli, Maurizio Stella, Anna Sapino, Carlotta Castagnoli

**Affiliations:** 1 Division of Breast Surgery, Department of General and Specialized Surgery, Città della Salute e della Scienza, Turin, Italy; 2 Division of Pathology, Department of Medical Sciences, University of Turin, Turin, Italy; 3 Skin Bank, Department of General and Specialized Surgery, Città della Salute e della Scienza, Turin, Italy; 4 Cytoimmunodiagnostic Laboratory, Department of Public Health and Pediatrics, University of Turin, Turin, Italy; 5 Fondazione del Piemonte per l’Oncologia (FPO) – Candiolo Cancer Institute (IRCCs), Candiolo, Italy; Medical University of South Carolina, UNITED STATES

## Abstract

Human Acellular Dermal Matrices (HADM) are employed in various reconstructive surgery procedures as scaffolds for autologous tissue regeneration. The aim of this project was to develop a new type of HADM for clinical use, composed of glycerolized reticular dermis decellularized through incubation and tilting in Dulbecco’s Modified Eagle’s Medium (DMEM). This manufacturing method was compared with a decellularization procedure already described in the literature, based on the use of sodium hydroxide (NaOH), on samples from 28 donors. Cell viability was assessed using an MTT assay and microbiological monitoring was performed on all samples processed after each step. Two surgeons evaluated the biomechanical characteristics of grafts of increasing thickness. The effects of the different decellularization protocols were assessed by means of histological examination and immunohistochemistry, and residual DNA after decellularization was quantified using a real-time TaqMan MGB probe. Finally, we compared the results of DMEM based decellularization protocol on reticular dermis derived samples with the results of the same protocol applied on papillary dermis derived grafts. Our experimental results indicated that the use of glycerolized reticular dermis after 5 weeks of treatment with DMEM results in an HADM with good handling and biocompatibility properties.

## Introduction

Human Acellular Dermal Matrices (HADM) provide a clean scaffold for host cellular and vascular in-growth. They are used in various reconstructive surgery procedures, for tissue regeneration in full thickness burns and for suspension, reinforcement and ligamentous restoration in breast reconstruction, abdominal wall repair and capsular repair in orthopedic surgery, respectively [1–4]. HADM provide biomechanical support and elicit new tissue formation by interacting with host cells through the modulation of cellular behaviors such as adhesion, migration, proliferation and differentiation. Furthermore, they modulate the inflammatory response and the remodeling of the newly formed extracellular matrix. However, the use of allograft dermis as a permanent tissue replacement is limited by its immunogenic properties [5]. In particular, the immune response is directed primarily against immunocompetent and structural cells in the epidermis and in the dermis [6,7]; on the opposite, it has been demonstrated that the extracellular matrix proteins are relatively non-immunogenic [8]. The evidence that freeze-dried bone allografts, in which only the extracellular matrix was preserved, did not elicit a specific immune response [9,10], led to the development of various techniques for xenologous [11] and human ADM production [12].

HADM manufacturing methods need therefore to preserve the mechanical characteristics of the tissue in order to maintain a structurally intact natural three-dimensional extracellular matrix able to integrate into the host tissue, but also need to produce a tissue as free as possible of potentially immunogenic antigens. The most common preservation methods used for skin dermo-epidermal allografts are cryopreservation and glycerolization. The latter is a simple and cost-effective method to produce non-viable but intact skin grafts that can be used as biological dressing or as temporary coverage on excised burns [13]. Glycerol preserved allografts lose their viability, whereas collagen and elastin fibers are well preserved, and the morphology of stromal and cellular components is well preserved [14]. In addition, glycerol 85% has a slow but effective virucidal and antiseptic action [15].

Commercially available HADM are structurally composed of the entire dermal layer. In the present study, we describe a new process for HADM production, based on the use of pure reticular dermis (RD) preserved by glycerol, in order to obtain dermal grafts suitable for clinical use. In fact, RD is relatively poor in cellularity, shows a strong mechanical resistance when compared to papillary dermis (PD) and has not basal membrane: all of these features could facilitate the re-colonization of grafts by host cells [16]. However, decellularization remains a mandatory step for ADM production in order to obtain a non-immunogenic matrix.

Several methods of decellularization exist; each of them is different in terms of duration, host cells’ re-population ability and alteration of the proteins and structures of the scaffold. The most commonly used include a combination of physical (e.g., freezing and thawing, tilting, sonication and γ ray irradiation), chemical (e.g., alkaline and acid treatments, ionic, non-ionic and zwitterionic detergents) and enzymatic (trypsin, endonucleases and exonucleases) approaches [17].

The first aim of our study was to evaluate the effectiveness of reticular dermal matrix decellularization obtained by using the Dulbecco’s Modified Eagle’s Medium (DMEM), a non chemical decellularization method, as compared with the NaOH chemical method [18–20].

We than evaluated the reproducibility and reliability of DMEM for obtaining HADM, comparing RD and PD samples.

## Materials and Methods

This project was carried out according to a study protocol on the use of an HADM composed of glycerolized RD decellularized for breast reconstruction, approved by the Institutional Ethical Committee (Comitato Etico Interaziendale A.O.U. San Giovanni Battista di Torino—A.O. “C.T.O. Maria Adelaide di Torino”) of Azienda Ospedaliera Universitaria Città della Salute e della Scienza of Turin, Italy, on January 23^rd^ 2012 with protocol number 0006730.

The Ethical Committee waived the need for a specific consent to the use of human dead donors tissues because, according to the Italian legislation on gathering of human dead donor organs and tissues, all citizens have to declare whether they do not want to be donors, otherwise they are automatically considered to have given their consent for donation (Ministry of Health, Legge 1 aprile 1999, n.91—Disposizioni in materia di prelievi e di trapianti di organi e di tessuti; Art.4 –Dichiarazione di volontà in ordine alla donazione). In addition, the Turin Skin Bank (www.cittadellasalute.to.it/?option=com_content&view=article&id=1881%253Abanca-della-cute-di-torino&catid=38&Itemid=1) furthermore provides a non-dissent form, which has to be signed by first line parents of dead donors.

On November 18^th^ 2011, the National Transplant Centre formally authorized the Turin Skin Bank to process, store and provide specimens from the back of 28 dead multiorgan donors who were unfit for transplantation.

### Preparation of the glycerolized human reticular dermal grafts

Specimens from the backs of 28 dead multiorgan donors were collected by the Turin Skin Bank after the harvesting of split thickness skin grafts assigned to be processed as allografts for the temporary coverage of burns. Human RD grafts of different thicknesses (0.4, 0.6 and 1.0 mm) were harvested and processed in the laboratory of our Skin Bank.

In particular, strips of specimens were dissected along the cranio-caudal direction and the same orientation was maintained during sampling.

In addition, samples of skin grafts of different thicknesses (0.4, 0.6 and 1.0 mm), glycerolized and de-epithelized, were used as samples of PD derived grafts, as described in literature [21].

Dermal grafts were placed in a medium composed of glycerol at a concentration of 50%, amikacin (1 mg/ml) and ampicillin (600 μg/ml) and then incubated for at least three hours at room temperature. The glycerol concentration was subsequently increased to 70% and then to 85%. The allografts were agitated gently for at least three hours at 33°C at each step.

After glycerolization, the dermal grafts were trimmed, measured and sealed in labelled plastic boxes, which were stored at a temperature of 4°C at least for 2 weeks. Dermal grafts were then de-glycerolized by sequential washing in a sterile 0.9% NaCl solution at 37°C.

### Decellularization protocols of human dermal grafts

RD derived grafts were incubated in two different media: DMEM (Biowest SAS, Nuaillé, France) or NaOH 0.06 N (Sigma-Aldrich, St. Louis, MO, USA) at room temperature in continuous tilting [22]; PD derived grafts were incubated in DMEM only. Samples were incubated in the specific medium for 8 weeks, changing the medium once a week. Grafts incubated with NaOH were neutralized by means of incubation in HCl 0.1 N before the analysis.

### Microbiological tests

Samples were placed in a broth-based medium (heart/brain) and sent to the Microbiology Laboratory, where they were incubated for 7 days at 35°C. Two subcultures were carried out on blood agar, the first one after 24 hours of incubation and the second at 7 days, to identify any contamination of slow-growth microorganisms. The samples were studied to isolate aerobic microorganisms and/or fungi. The analyses were performed three times for each sample according to Good Manufacturing Practice (GMP) guidelines: 1596 samples obtained from 28 donors were analyzed. Microbiological monitoring was performed on all samples processed after each step: immediately after harvesting, after decontamination, after glycerolization and after each change of medium during decellularization.

### Evaluation of dermal viability on reticular dermis

The tetraziolium salt (MTT) test was performed on human RD graft samples (n = 1680) to assess cell viability immediately after harvesting (fresh), after glycerolization (T0) and then at each one-week time interval during the decellularization processes. The technique was set up using dermal disks obtained by punch-biopsies (Stiefel Laboratories, Sligo, Ireland) and placed in a 24-well tissue culture plate. Six punches with similar weights, including 2 negative controls, were chosen and tested for each specimen. MTT salts (0.5 mg/ml) were added to RPMI without antibiotics. The samples were then incubated at 37°C in 5% CO_2_. After 3 hours of incubation the precipitated salts were solubilized for 3 hours with the use of 2-methoxyethanol (Sigma-Aldrich, St. Louis, MO, USA). The solution was then read on a spectrophotometer (570 nm). A negative control was always set up in duplicate (a heat-denatured specimen after 20 minutes of microwave at maximum temperature) for each experimental condition and its optical density (OD) was subtracted from the OD of each sample.

The tissue viability of each sample was expressed as the ratio between the OD of the dermis and its weight in grams.

$$Viability\;Index=\frac{OD(570nm)}{grams\;of\;tissue}$$

### Macroscopic evaluation of the dermal grafts

Macroscopic evaluation of the dermal grafts was carried out by clinical examination in order to assess some of the mechanical and handling characteristics.

Six 4x4 cm dermal grafts derived from RD, three decellularized using NaOH and three using DMEM, were placed on a surgical shelf. Each of the respective triplets showed different characteristics in terms of the decellularization protocol, while the thickness and incubation time were identical. Grafts A, B and C were treated with NaOH while D, E and F with DMEM. The thicknesses were 0.4 mm (A and D), 0.6 mm (B and E) and 1 mm (C and F) and the incubation time was 5 weeks for all samples.

Characteristics of the dermal grafts evaluated were elasticity, pliability, tear and needle penetration resistance. A numeric rating scale ranging from 1 to 5 (1 corresponding to poor, 2 to sufficient, 3 to average, 4 to good and 5 to excellent) was used to evaluate each characteristic. Two independent surgeons [surgeon 1 (S1) and surgeon 2 (S2)] were asked to evaluate the characteristics of the dermal grafts by manipulating and sewing the samples in a double blind fashion. To subjectively evaluate elasticity and tear resistance, the surgeons were asked to pull the grafts in opposite directions with surgical forceps; pliability and needle penetration resistance were assessed by placing the grafts on a 1x1 hemispherical silicon skin expander valve (Allergan, Marlow International, Buckingamshire, UK) and by sewing their perimeter with a sharp needle 2/0 PDS (Ethicon, Somerville, NJ, USA) suture with simple stitches.

The same macroscopic evaluation described above was performed on six 4x4 cm dermal grafts decellularized using DMEM, three derived from PD and three from RD, which were placed on a surgical shelf. Each of the respective triplets showed different characteristics in terms of the dermal layer origin, while the thickness and incubation time were identical. Grafts G, H and I were made of PD while J, K and L were made of RD. The thicknesses were 0.4 mm (G and J), 0.6 mm (H and K) and 1 mm (I and L) and the incubation time was 5 weeks for all samples.

Data were collected and statistically compared (Spearman’s *rho* and Kendall’s *tau*-B coefficients analysis of the results). A p-value less than 0.05 was considered statistically significant.

### Tissue Specimens and Staining Procedure

A first biopsy was taken from every dermal matrix sample upon arrival of the specimens in the laboratory and used as a fresh control; a second biopsy was taken after the glycerolization procedure for use as a non-treated control (T0). A biopsy was then taken at every week of treatment, from week 1 (T1) to week 8 (T8) after incubation in the specific medium (NaOH or DMEM). Biopsy samples were then washed in physiologic solution, fixed in 4% neutral-buffered formalin at room temperature and embedded in formalin by routine processing (FFPE). FFPE samples were sectioned at a thickness of 2–3 μm for histochemistry and immunohistochemistry reactions. For standard histology Hematoxilin-Eosin (HE) staining was performed with the automatic Leica ST5020 stainer. Immunohistochemistry was performed using an automated slide-processing platform (Ventana BenchMarckXT Autostainer, Ventana Medical Systems, Tucson, AZ, USA). Antibody clones, dilution and antigen retrieval are shown in Table 1.

**Table 1:** Antibodies used for immunohistochemical reactions.

Antibody	Clone	Species	Manufacturer	Dilution	Antigen Retrieval	Primary Ab^a^ Incubation
Laminin	4C7	Mouse	Dako	1:20	Protease 4'	32' at RT^c^
Collagen IV	CIV 22	Mouse	Dako	1:50	CC1^b^ 30'	30' at RT^c^
Vimentin	V9	Mouse	Dako	1:150	CC1^b^ 30'	16' at 37°C
CD31	JC70A	Mouse	Dako	1:50	CC1^b^ 30'	32' at 37°C
CD34	QBEnd/10	Mouse	Roche-Ventana	Prediluted	CC1^b^ 30'	24' at 37°C
CD45/CLA	2B11 + PD7726	Mouse	Dako	1:50	/	20' at RT^c^
CD68	KP1	Mouse	Dako	1:50	Protease 4'	24' at 37°C

^a^antibody;

^b^cell conditioning;

^c^room temperature

To investigate the characteristics of the interstitial matrix and the basement membrane, collagen and elastin fibers were stained using trichrome (Gomori’s green Trichrome stain, DakoArtisanLink automatic stainer), elastic van Gieson (EVG, DakoArtisanLink automatic stainer) and sirius red (SiriusRot F3BA—Chroma Gesellchaft, manual procedure) histochemical reactions and by immunohistochemistry with antibodies directed against laminin, collagen IV and vimentin. The orientation, medium length and thickness of the fibers were assessed at forty times magnification (40x).

To study the cellular components of the dermal matrix, antibodies directed against vimentin, CD31 and CD34, CD45/CLA and CD68 were used. Variations in the amount of the cellular components at different time points during tissue treatment with DMEM or NaOH were scored using a grid with the ocular of the microscope measuring 1 mm^2^ (10 by 10 fields) at an objective magnification of twenty times (20x), considering the intensity of the staining (from + to +++) and the distribution of the positive elements (focal *versus* diffuse). Two independent observers analyzed the sections.

### DNA extraction in reticular dermis samples

A total of 170 samples (T0-T8) obtained from 10 donors were analyzed in this study; 50 mg of each dermal specimen at different incubation times were incubated overnight at room temperature with 500 μl of lysis buffer (100 mM NaCl, 10 mM TrisHCl pH8, 1 mM EDTA pH8, 1% SDS, 2% Triton X-100). Tubes were incubated at 100°C for 10 minutes. Equal volumes of phenol-chloroform were added to the samples and the tubes were centrifuged at 13000 rpm for 10 minutes. Upper aqueous phase transfer into fresh tubes was followed by the addition of 1 vol of ice cold ethanol, incubated at -20°C for 30 minutes, then centrifuged at 12000 rpm for 10 minutes at 4°C. DNA pellets were separated from supernatants. Pellets were washed twice with 70% ethanol, centrifuged at 10000 rpm for 10 minutes then dried at room temperature from the tubes. Pellets were dissolved in 20 μl of distilled water. The DNA samples were quantified with a NanoDrop^™^ 1000 spectrophotometer and stored at −20°C until they were assayed.

### Genomic DNA quantification with real-time TaqMan MGB probe in reticular dermis samples

For DNA quantification we used an approach first described in forensic samples [23]. Genomic DNA obtained from peripheral blood mononuclear cells (PBMC) was used to set up a standard curve as follows. Using 100 ng as the initial concentration, five dilutions were performed by making 1:10 dilution series. Each standard dilution was tested three times to ensure repeatability. Primers and probe were designed with primer express software version 3.0 (Applied Biosystems, Waltham, MA, USA) and found to be unique for glyceraldehyde-3-phosphate dehydrogenase (GAPDH) after ‘‘blastn” (http://www.ncbi.nlm.nih.gov/BLAST/). Specific sequences for GAPDH primers were GAPDHF-5’-CCAAGGTCATCCATGACAAC-3’ and GAPDHR-5’- GTGGCAGTGATGGCATGGAC-3’; the sequence for the GAPDH probe was GAPDH-6FAM- TGGTATCGTGGAAGGA-3’.

For absolute quantification with the real-time TaqMan MGB probe, 5 μl of 1/50 DNA diluted samples obtained from dermal matrices at different incubation times were added to a 2X Applied Biosystem “ready to use” master mix (Life Technologies, Carlsbad, CA, USA) with 500 mM of primers and 150 nM of probe and run in PCR under the same conditions as the standards. Wells with no DNA served as no template controls. The amplification conditions used were 95°C for 10 minutes, followed by 40 cycles of 95°C for 15 s and 60°C for 1 minute.

Absolute real-time PCR data were analyzed with SDS 2.4 Software (Applied Biosystems). All analyses were based on the Ct values of the PCR products, which are the PCR cycles at which the fluorescence measured between each cycle exceeds a threshold determined by background fluorescence at baseline and is placed in the exponential phase of the amplification curve. The SDS software set the baseline automatically and calculated the standard curve for each run based on the Ct for the standard. To evaluate the residual amount of DNA in the dermal matrices tested, the slope (s) and the y-axis intercept (Y) (the y-axis intercept is the point at which the standard curve intersects with the ordinate; it indicates the theoretical detection limit of the reaction by revealing the Ct expected in the presence of a single target molecule in the sample) of the corresponding standard curve and the Ct of the target amplification were used according to the following equation:

$$Po=Inverse\text{ log}\left(Ct-\frac{Y}{s}\right)$$

Po is the number of ng equivalents in the PCR prior to amplification.

## Results

### Microbiological tests

All samples were sterile after glycerolization and decellularization processes, even though 55% of samples were contaminated after harvesting.

### Evaluation of dermal viability on reticular dermis

**Fig 1:**
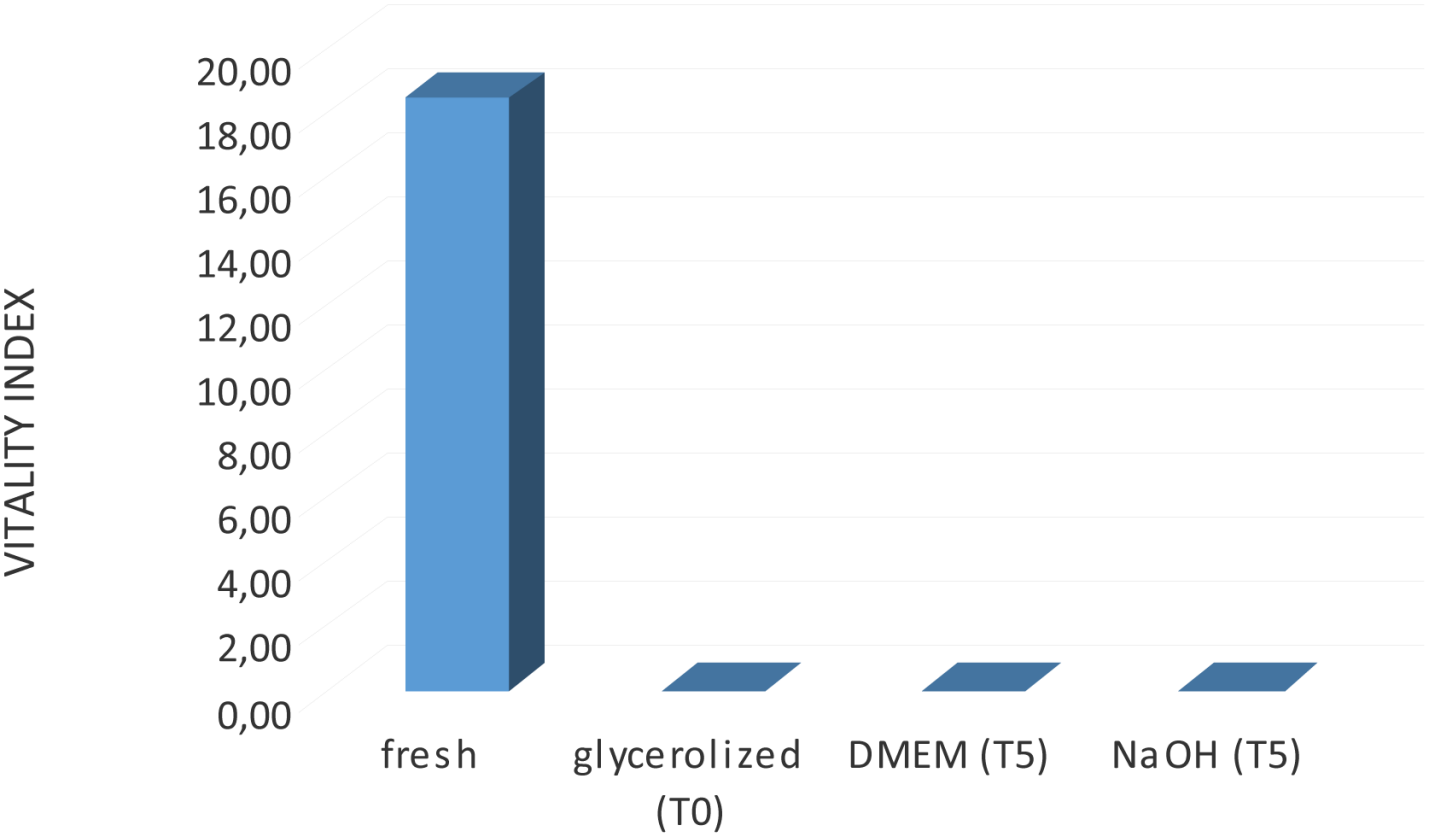
MTT test for evaluation of dermal viability on reticular dermis. Results of the vitality index assessment showed the absence of viable cells in the HADM after glycerolization and decellularization processes.

MTT quantitative analysis was carried out on the grafts to determine viability. Each graft’s viability was compared to that of the fresh dermal sample. The term “fresh sample” indicates a specimen analyzed immediately after harvesting and used as a positive control. The results showed the absence of viable cells in the processed grafts for both decellularization techniques (Fig 1).

### Quantification of DNA in reticular dermal grafts

DNA was successfully extracted, amplified and quantified in all the samples obtained from the dermal grafts.

**Fig 2:**
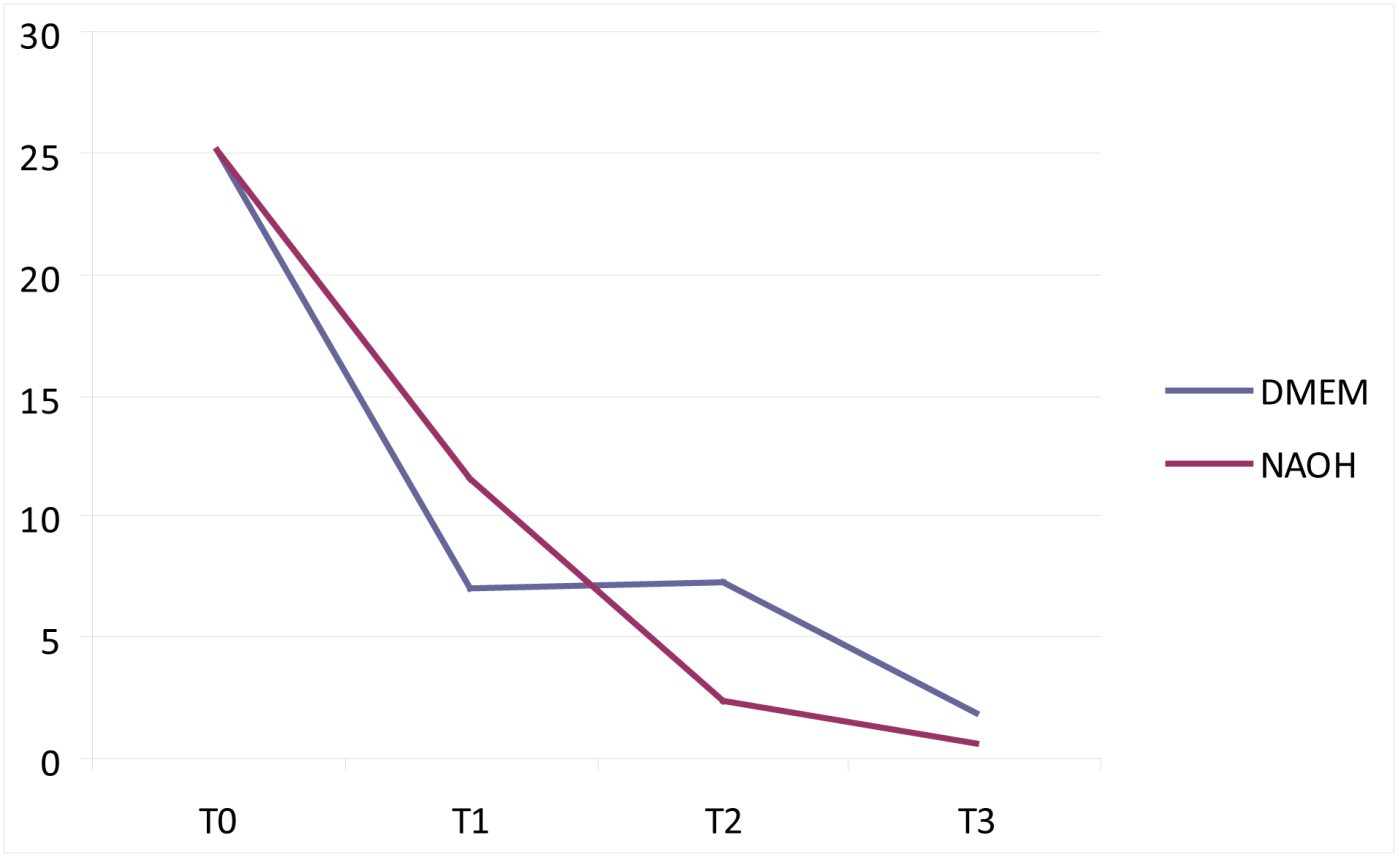
Quantity of DNA in 1 mg of tissue obtained from RD matrices at different incubation times using different decellularization protocols. The amount of DNA is reported on the y-axis (ng/mg) and the time of incubation is reported on the x-axis (from T1 to T3), using different colors referring to different decellularization methods (blue for DMEM and red for NaOH).

**Fig 3:**
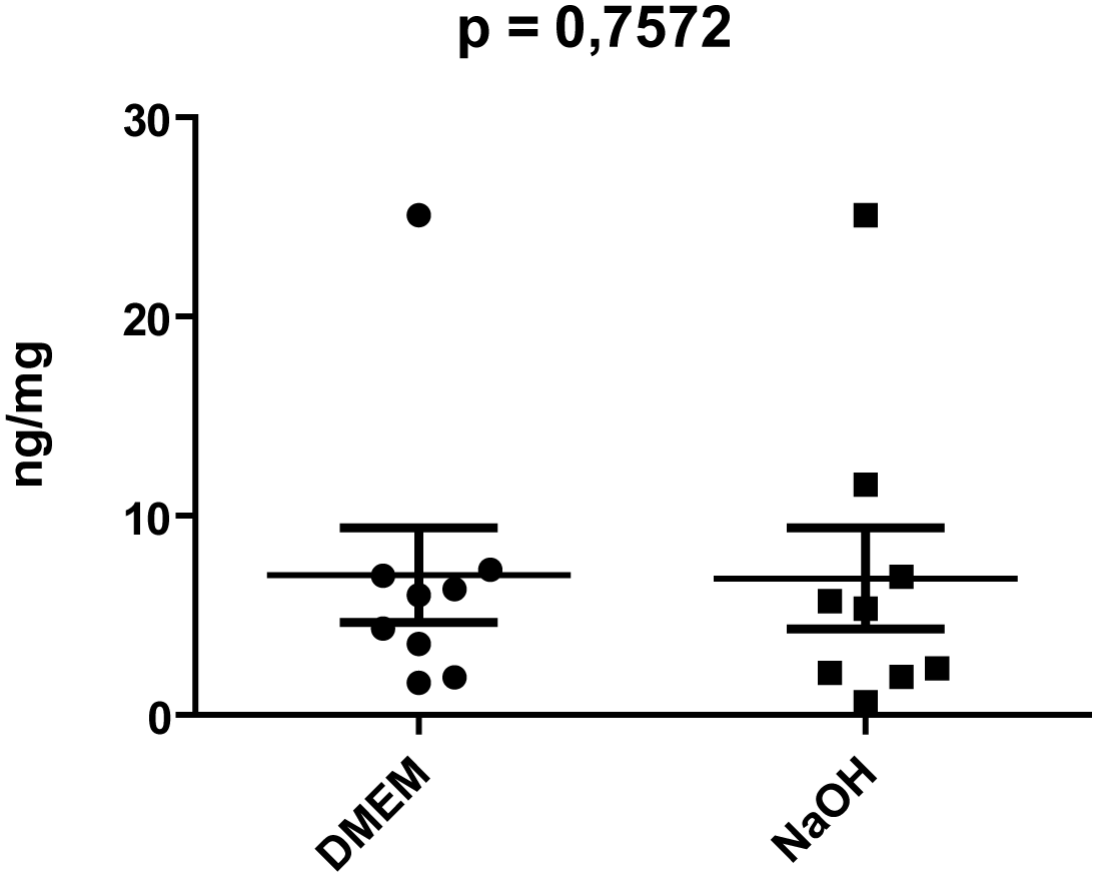
Mann-Whitney test. The amount of residual DNA from decellularized RD matrices was not significantly different using DMEM and NaOH.

The DNA content was quantified using a real-time TaqMan MGB probe assay and calculated as ng/mg tissue weight. Compared to unprocessed dermis, at least 81% of DNA content was removed through processing. The amount of DNA residues decreased until T3 and then remained constant up to T8, with minimal variations depending on the samples (Fig 2). There was no significant difference between treatment with DMEM and NaOH regarding the amount of DNA residues (p = 0.7209) (Fig 3).

### Performance of different decellularization methods on reticular dermis: macroscopic evaluation

Grafts incubated with the two different protocols were similar in terms of consistence and texture. When compared, the dermal grafts treated with NaOH 0.06N appeared more rigid and less flexible than those treated with DMEM, which, on the other hand, appeared to be smooth and elastic.

The scores of the clinical subjective macroscopic evaluation of the dermal grafts at 5 weeks (T5) performed by the two independent surgeons (S1 and S2) are shown in Table 2.

**Table 2:** Evaluation scores of biomechanical parameters of reticular dermis grafts of increasing thickness treated for 5 weeks with NaOH or DMEM, assessed by two surgeons (S1 and S2).

HADM characteristics	Samples treated with NaOH						Samples treated with DMEM					
	A (S1)	A (S2)	B (S1)	B (S2)	C (S1)	C (S2)	D (S1)	D (S2)	E (S1)	E (S2)	F (S1)	F (S2)
**Elasticity**	3	3	2	2	2	2	5	5	4	4	4	5
**Pliability**	4	4	4	3	2	2	4	4	4	5	4	4
**Tear resistance**	3	3	5	4	5	5	4	4	5	5	5	5
**Needle penetration resistance**	3	3	2	2	2	1	4	3	4	4	5	5
**Total Score**	13	13	13	11	11	10	17	16	17	18	18	19

There was good agreement between the two surgeons in the evaluation of the parameters (*rho* = 0.841; *tau-B* = 0.847; both p < 0.01), with the exception of pliability (*rho* = 0.645; *tau*-B = 0.696; both with p > 0.05).

In specimens treated with NaOH, the elasticity, pliability and needle penetration resistance scores decreased with increasing specimen thickness. In contrast, in specimens treated with DMEM the scores of these three parameters were higher in general than those of specimens treated with NaOH. Tear resistance increased with increasing specimen thickness. In general, the specimens treated with DMEM showed better performance and lower score variation, specifically the 1 mm thickness that showed the highest scores.

### Performance of different decellularization methods on reticular dermis: microscopic evaluation

The effectiveness of the decellularization of grafts was assessed by histology and immunohistochemistry; the reactions were performed on samples collected from weeks 1 to 8 (T1-T8), using fresh and T0 samples as controls. Immunohistochemical results are summarized in Table 3.

**Table 3:** Immunohistochemical results of decellularization obtained by the DMEM and NaOH protocols.

Staining	Fresh tissue	T0	DMEM							NaOH						
			T1	T2	T3	T4	T5	T6	T8	T1	T2	T3	T4	T5	T6	T8
Laminin	+++	+++	++	++ focal	++ focal	++ focal	+ focal	+ focal	+ focal	+ focal	+ focal	+ focal	-	-	-	-
Collagen IV	+++	+++	+++	+++	+++	+++	+++	+++	++	+++	+++	+++	++	++	++	++
CD31 and CD34	+++ diffuse	+++ diffuse	+++ diffuse	++ diffuse	++ diffuse	++ focal	+ focal	+ focal	+ focal	+ focal	+ focal	-	-	-	-	-
Vimentin	+++ diffuse	+++ diffuse	++ focal	++ focal	+ focal	-	-	-	-	+ focal	+ focal	-	-	-	-	-
CD45/CLA	+++	+++	+ focal	+ focal	+ focal	-	-	-	-	+ focal	-	-	-	-	-	-
CD68	+++	+++	++ focal	++ focal	+ focal	-	-	-	-	+ focal	-	-	-	-	-	-

#### Morphology on fresh tissue and glycerolized samples of reticular dermis before decellularization

**Fig 4:**
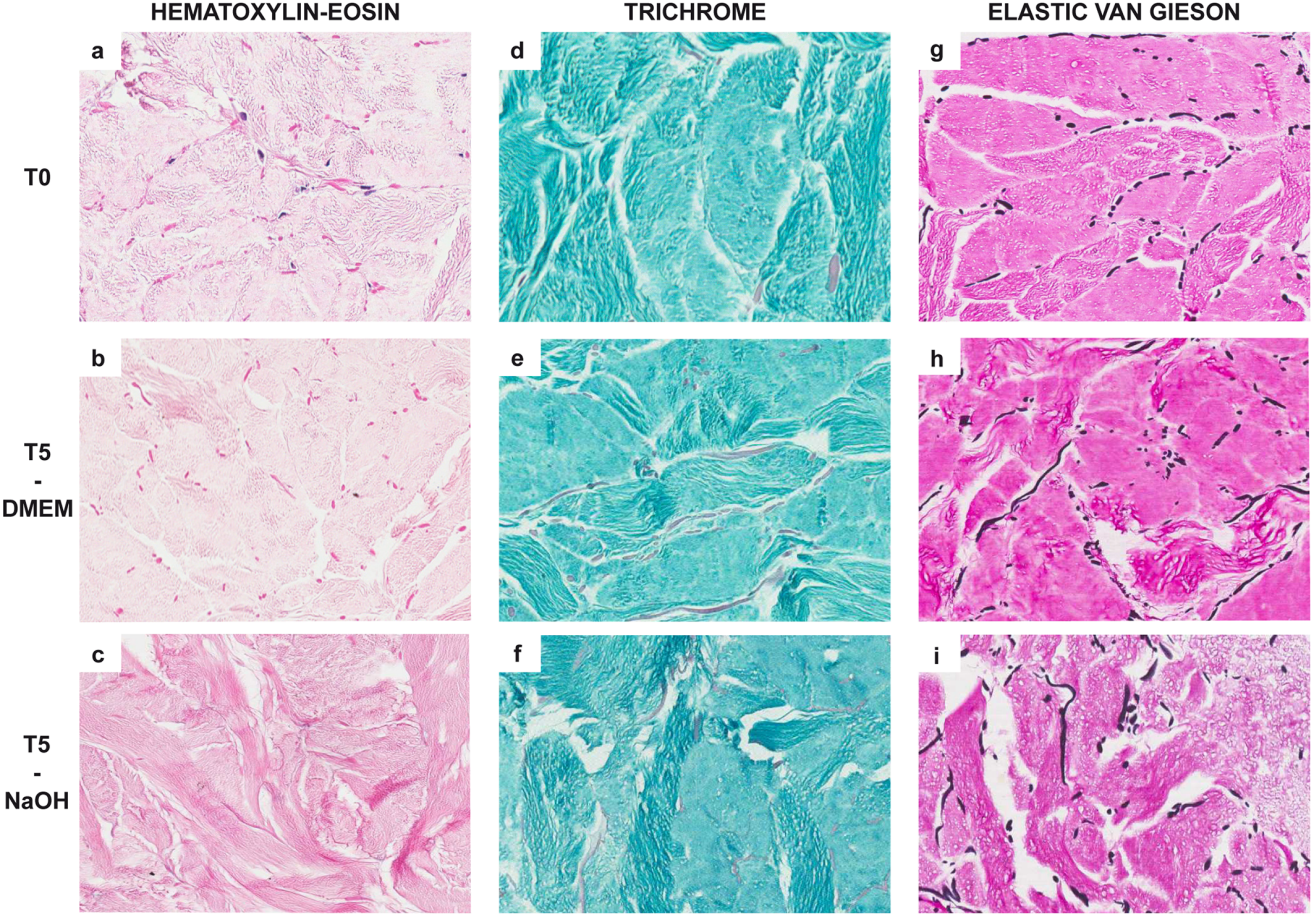
Histochemical reactions showing histological features of human acellular reticular dermal matrices at different incubation times (20X magnification). In comparison with control (T0) samples (**A**: hematoxylin-eosin; **D**: trichrome staining), after 5 weeks of treatment (T5), specimens showed the presence of stromal shrinkage and tissue fragmentation, edema and focal condensations with spongy patterns; these denaturation artifacts were rare in samples treated with DMEM (**B**: hematoxylin-eosin; **E**: trichrome staining) and more evident in those treated with NaOH (**C**: hematoxylin-eosin; **F**: trichrome staining). Elastic fibers, stained in black using Elastic Von Gieson histochemical reaction, showed no significant alterations in terms of length, diameter and mean number comparing T0 samples (**G**) with T5 specimens treated with DMEM (**H**) or NaOH (**I**).

The absence of epidermis and pars papillaris of the dermis was confirmed both on fresh tissue and on T0 samples. RD consisted of a three-dimensional meshwork of thin collagen bundles, as shown by HE (Fig 4A) and trichrome (Fig 4D) staining and confirmed by sirius-red staining under polarized light. EVG staining showed thick interconnected elastic fibers intermingled within the collagen fibers and arranged in a similar three-dimensional pattern (Fig 4G). The collagen and elastic fibers might display a different arrangement (long bundles or roundish segments), depending on the orientation of samples during cutting.

**Fig 5:**
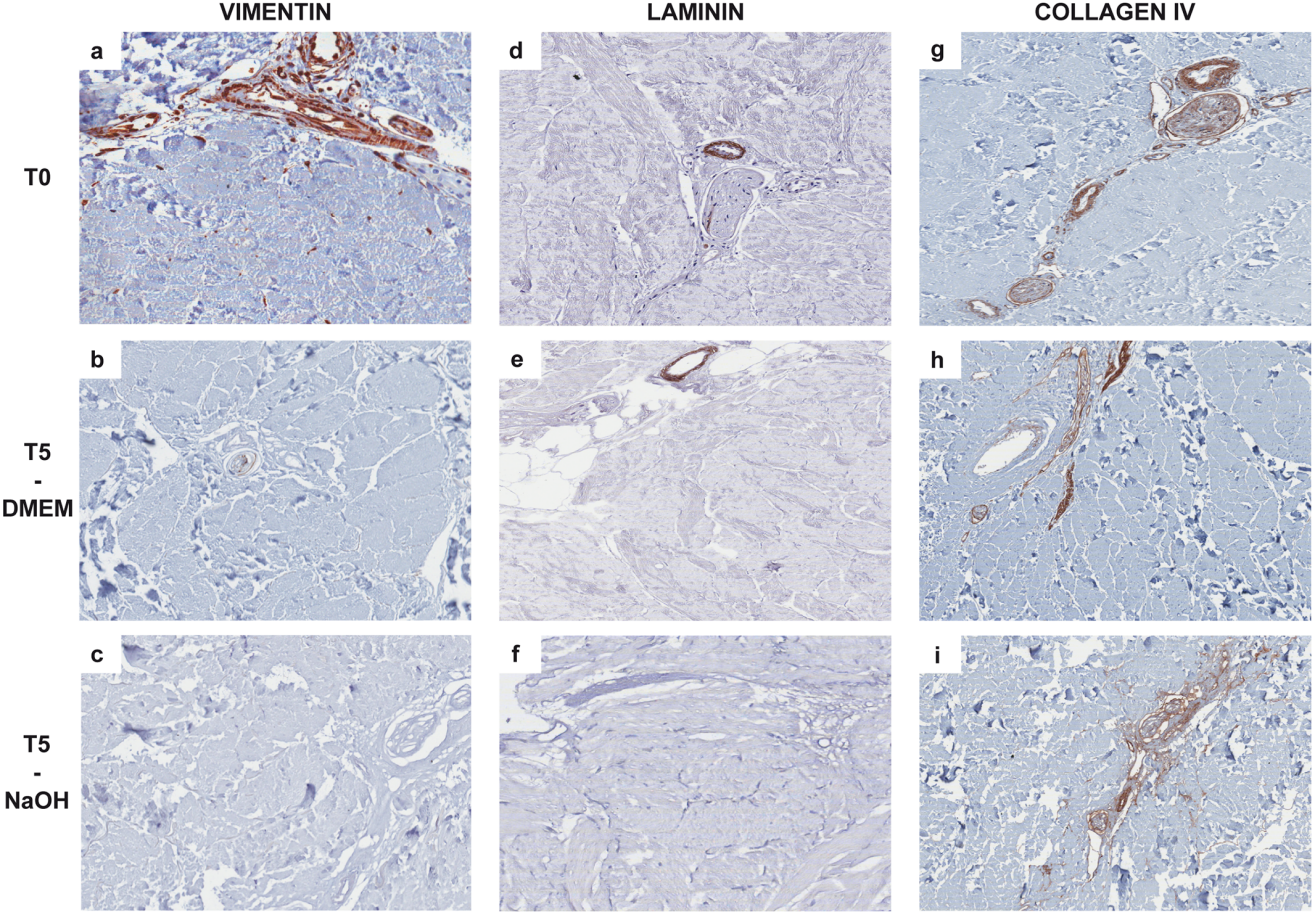
Immunohistochemical reactions showing histological features of the non-cellular component of reticular dermal matrices at different incubation times (20X magnification). In control samples (T0) vimentin (**A**), laminin (**D**) and collagen IV (**G**) immunohistochemical reactions showed the presence of an intact basement membrane (stained in brown), which was progressively degraded during the decellularization process; in DMEM treated samples, the immunostaining was focal and weak after 5 weeks (T5) of treatment (**B**: vimentin; **E**: laminin; **H**: collagen IV). Basement membrane degradations appeared more evident in T5 samples treated with NaOH, in which vimentin (**C**) and laminin (**F**) reactions were negative, and only collagen IV staining was still positive (**I**). Vimentin immunostaining also showed that both DMEM and NaOH were able to completely eliminate fibroblasts at T5.

Vimentin (Fig 5A), laminin (Fig 5D) and collagen IV (Fig 5G) immunostaining highlighted the presence of an intact basement membrane surrounding the vessels, adnexa and nerves.

**Fig 6:**
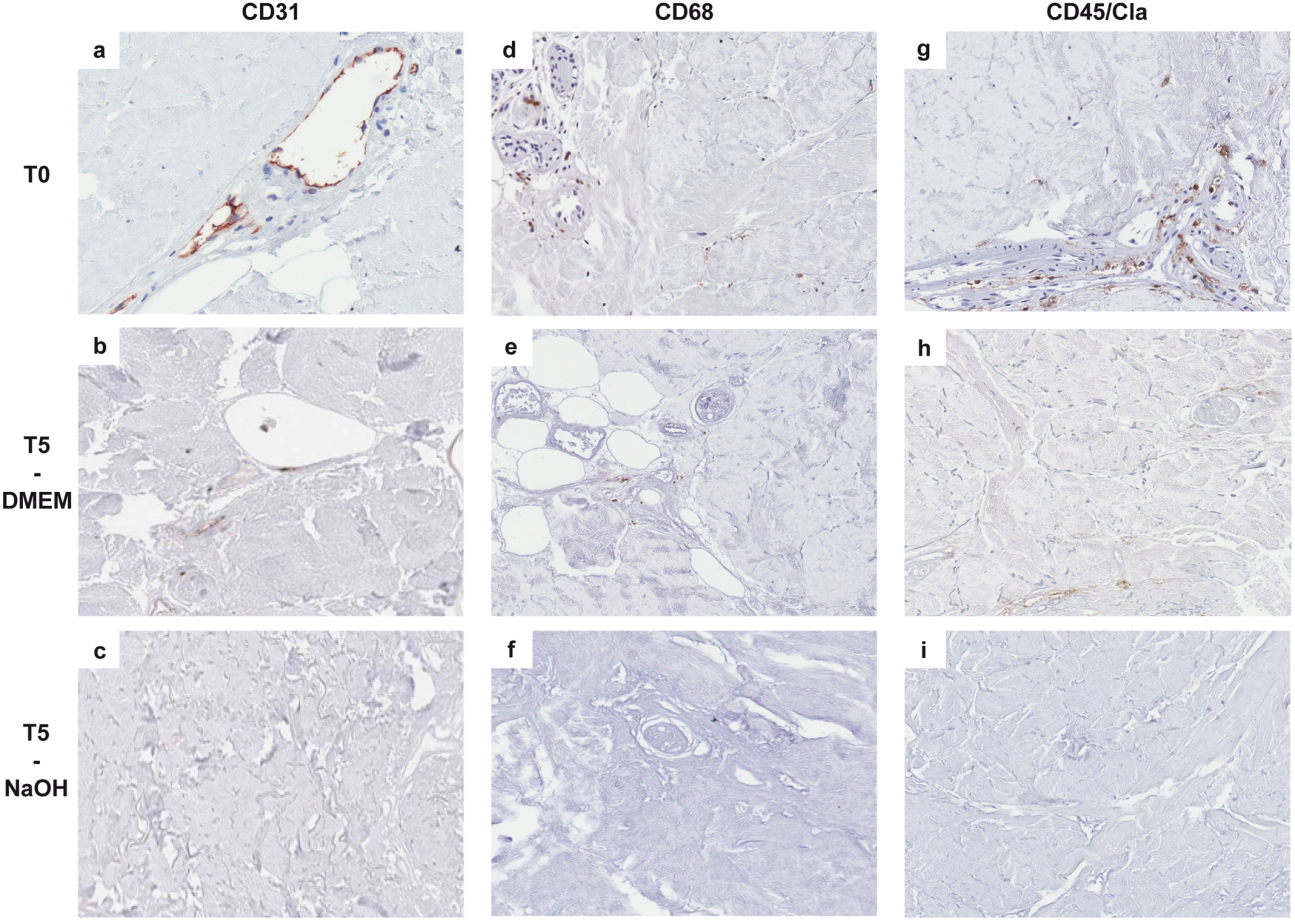
Immunohistochemical reactions showing histological features of the cellular component of reticular dermal matrices at different incubation times (20X magnification). In control samples (T0), the CD31 immunohistochemical reaction showed the presence of intact vessels (**A**; endothelial cells are stained in brown), while immunostaining was focal and weak after 5 weeks (T5) of treatment with DMEM (**B**) and completely absent in NaOH treated samples (**C**). Immunohistochemistry also showed that at T0 both macrophages (**D**, showing CD68 positive cells) and lymphocytes (**G**, showing CD45/CLA positive cells) were present, concentrated in perivascular spaces and near the residual lower portion of hair follicles, while only non-specific focal and weak staining was present after 5 weeks (T5) of treatment with DMEM (**E**, **H**); reactions were completely negative in NaOH-treated samples (**F**, **I**).

Immunohistochemistry also showed that fibroblasts (Fig 5A), vessels (Fig 6A), histiocytes, macrophages (Fig 6D) and lymphocytes (Fig 6G) were present in relatively small numbers; lymphocytes were more concentrated in the perivascular spaces and near the residual lower portion of hair follicles.

#### Non-cellular components on reticular dermis at different times of treatment using different decellularization techniques

HE and trichrome staining showed that on the short period specimens had structural morphology unchanged compared to T0 for both decellularization techniques (DMEM and NaOH), while longer exposure to both compounds led to denaturation artifacts. In particular, stromal shrinkage with consequent tissue fragmentation, presence of edema and focal condensations with a spongy pattern were observed after 5 weeks, but were more evident in NaOH-treated samples (Fig 4B, 4C, 4E and 4F).

Elastic fibers similarly did not show significant structural alterations during decellularization. Independently from the duration of the treatment and the decellularization technique used, elastic fibers measured from 10 μm to 800 μm, with an average diameter of 7.5 μm; this number remained consistent (Fig 4G, 4H and 4I). All of the specimens showed a random orientation of fibers within the collagen; however, at T6-T8 the orientation became more chaotic as a consequence of tissue fragmentation.

These results were confirmed by sirius red staining using polarized light; collagen fibers did not show substantial remodeling and preserved their natural double refraction from T0 to T8.

We observed a progressive degradation of the basement membrane during the decellularization process using both DMEM and NaOH (Fig 5); nevertheless, it was much more evident in samples treated with NaOH, in which only collagen IV staining was still positive after 5 weeks of treatment.

#### Cellular components on reticular dermis at different times of treatment using different decellularization techniques

Immunohistochemical reactions showed that both DMEM and NaOH were able to completely eliminate fibroblasts (Fig 5B and 5C), macrophages/dendritic cells (Fig 6E and 6F) and lymphocytes (Fig 6H and 6I) from the specimens. Using NaOH the decellularization was achieved in a shorter period of time, from T2 to T3, compared to DMEM, which produced the same results after 4 weeks.

NaOH was able to completely eliminate the endothelial cellular component after two weeks whereas DMEM showed a progressive reduction in vessels from week four (T4) but residual CD31 immunostaining was still present at T5 (Fig 6A–6C) and after 8 weeks of treatment (Table 3).

### DMEM decellularization protocol on different types of dermis: macroscopic comparison

Grafts derived from PD and RD incubated with the DMEM decellularization protocol were similar in terms of consistence and texture.

The scores of the clinical subjective macroscopic evaluation of the dermal grafts at 5 weeks (T5) performed by the two independent surgeons (S1 and S2) are shown in Table A in S1 File. There was good agreement between the two surgeons in the evaluation of the parameters (*rho* = 0.768; *tau-B* = 0.735; both p < 0.01).

PD grafts appeared more resistant to tearing and needle penetration and less pliable and elastic than the RD ones, in particular for the thinnest (0.4 mm) of the three thicknesses assessed. No other differences in the biomechanical parameters analyzed were highlighted between the two types of dermis; they showed overall analogous performance and score variation, specifically in samples of 1 mm of thickness.

### DMEM decellularization protocol on different types of dermis: microscopic comparison

The effectiveness of the DMEM decellularization protocol on RD and PD derived grafts was assessed by histology and immunohistochemistry; the reactions were performed on fresh samples, on glycerolized samples (T0) and on samples treated with DMEM at weeks 1 (T1) and 5 (T5). Immunohistochemical results are summarized in Table B in S1 File.

On fresh tissue and glycerolized samples of PD before decellularization (T0), HE, trichrome and siurius-red staining showed a frame made of collagen fibers and thin bundles, with reticulum fibers present in a dense array. EVG staining showed that the medium length of elastic fibers was consistent with that of the RD; nevertheless, in PD we observed a higher amount of fibers, with an average diameter of 15 μm. As expected, PD showed a higher amount of vessels compared with RD.

During treatment with DMEM, HE and trichrome staining performed on PD derived grafts did not show significant differences in comparison with RD derived samples at each time of decellularization. On the short period (T1) specimens had structural morphology unchanged compared to T0 for both RD and PD, while longer exposure to DMEM led to mild denaturation artifacts. Elastic fibers similarly did not show significant structural alterations during decellularization both in RD and in PD derived grafts. All of the specimens showed a random orientation of fibers within the collagen. These results were confirmed by sirius red staining using polarized light.

The progressive degradation of the basement membrane observed during the decellularization process in RD derived samples was present in PD derived samples as well.

Immunoistochemistry showed that on fresh tissue and glycerolized samples before decellularization, PD contained relatively more cells (fibroblasts, histiocytes, macrophages and lymphocytes) compared with RD. Lymphocytes and histiocytes were more concentrated in the perivascular spaces and near residual adnexa. In PD, adnexa were mostly composed of complete hair follicles.

Treatment with DMEM did not completely eliminate fibroblasts, macrophages/dendritic cells and lymphocytes from PD derived grafts, as after 5 weeks (T5) weak and diffuse immunohistochemical staining for CD45/CLA and CD68 was observed. A progressive reduction in the amount of vessels was observed both in RD and in PD derived grafts; nevertheless, the number of residual vessels was considerably higher in PD samples after 5 weeks of treatment (Table B in S1 File).

## Discussion

Several methods of producing HADM are described in the literature, distinguished in terms of preservation, tissue origin and characteristics and decellularization procedures [17,20,22,24,25].

We used glycerolization as a preservation method for RD samples, a technique already described in human ADM production processes [13–15,18–20,25,26]. This method is cost-effective [13], produces non-viable sterile grafts without major damage of the extracellular matrix [14,15] and is easy to use for widespread clinical applications. The Turin Skin Bank has a consolidated experience in glycerolization as a method of producing temporary skin substitutes.

To our knowledge this is the first report of a dermal product entirely composed of RD that retains its intrinsic properties; RD constitutionally shows a low cellularity that in turn results in a reduced need for decellularization processes. In addition, the absence of the skin basal membrane, which is retained in other commercially available ADMs composed of papillary dermis, fosters *in vitr*o cell penetration into the extracellular matrix [16,25].

We compared the decellularization method based on the use of DMEM with the one based on the use of NaOH, both combined with mechanical tilting.

The goal of a decellularization protocol is to efficiently remove all cellular and nuclear material in order to obtain a non-immunogenic matrix, while minimizing any adverse effect on the composition, biological activity and mechanical integrity of the remaining extracellular matrix [17].

Physical methods require a long time but assure good preservation of proteins (such as laminin) and matrix integrity without residual cytotoxicity [17].

NaOH has already been used in the processing of human-derived tissues in order to remove donor cells and annexes [18–20,27]. In contrast, DMEM has been shown to be able to maintain the overall properties of the living tissue, promoting the mobilization of the structural cells [28].

In the present study, the immunohistochemical evaluation of both DMEM and NaOH showed a good decellularization of grafts after 4 weeks of treatment. The DNA absolute quantification was reduced by more than 90% at 3 weeks (Fig 2), and this residual DNA is considered to be unable to elicit any antigenic reaction [24]. In addition, the residual DNA content in other commercially available HADM is in general higher than that found in our matrix [24,26].

However, microscopic examination of the histochemical reactions demonstrated fewer artifacts (fragmentation, presence of edema and focal condensations with spongy patterns) of the tissue after 5 weeks of treatment with DMEM compared to NaOH. Nevertheless elastin and collagen fibers did not show substantial remodeling and preserved their natural double refraction features with both procedures. This has to be considered as an advantage because it has been hypothesized that the elastin fibers present in donor skin are not replaced, but they are used as guidance for the formation of new blood vessels and the migration of fibroblasts. This phenomenon results in more organized new collagen fiber deposition and higher pliability [18]. The laminin and collagen of the vascular endothelial elements were preserved as well. Their persistence has proven to be critical for autologous cell migration into the scaffold, serving as a modulator for cellular re-population of the dermal matrix (i.e., neodermis formation) [16].

Our results showed that grafts incubated with the two different media appeared similar in terms of consistency and texture; however, DMEM-treated samples were less rigid, more elastic and flexible according to surgeon evaluation, with the best performance observed at the 1 mm thickness. The long incubation time in media could favor bacterial growth. However, the microbiological monitoring of the samples indicated that the operative protocols used in our Skin Bank guarantee sterility.

Finally, we did not observe any significant difference, both macroscopically and microscopically, in using DMEM decellularization protocol on RD and PD derived grafts.

All of these results demonstrate the reliability and reproducibility of the non-chemical method using DMEM incubation and tilting for 5 weeks on RD to obtain a “Human Acellular Reticular Dermal Matrix” (HARDM). The DMEM protocol was more effective in preserving the architectural features of the extracellular matrix than NaOH chemical method.

Our results suggest that HARDM could be an ideal matrix for reconstructive surgery after pre-clinical *in vivo* validation.

## Supporting Information

S1 File Comparison between reticular and papillary dermis derived grafts. Evaluation scores of biomechanical parameters of reticular and papillary dermal grafts of increasing thickness treated for 5 weeks with DMEM, assessed by two surgeons (S1 and S2)**(Table A)**. Immunohistochemical results of decellularization of reticular and papillary dermis obtained using DMEM **(Table B)**.
(DOCX)
