# Supplementary material for: Glycerolized Reticular Dermis as a New Human Acellular Dermal Matrix: An Exploratory Study
Source: PLoS One. 2016 Feb 26;11(2):e0149124. doi: 10.1371/journal.pone.0149124 (PMC4769070; doi:10.1371/journal.pone.0149124)
Supplement: S1 File — Evaluation scores of biomechanical parameters of reticular and papillary dermal grafts of increasing thickness treated for 5 weeks with DMEM, assessed by two surgeons (S1 and S2)(Table A). Immunohistochemical results of decellularization of reticular and papillary dermis obtained using DMEM (Table B). (DOCX) [file pone.0149124.s001.docx]

**Table A.** **Evaluation scores of biomechanical parameters of reticular and papillary dermal grafts of increasing thickness treated for 5 weeks with DMEM, assessed by two surgeons (S1 and S2).**

| **HADM characteristics** | **RETICULAR DERMIS DERIVED SAMPLES** | | | | | | **PAPILLARY DERMIS DERIVED SAMPLES** | | | | | |
| --- | --- | --- | --- | --- | --- | --- | --- | --- | --- | --- | --- | --- |
|  | **G (S1)** | **G**  **(S2)** | **H (S1)** | **H (S2)** | **I (S1)** | **I (S2)** | **J (S1)** | **J (S2)** | **K (S1)** | **K (S2)** | **L (S1)** | **L (S2)** |
| **Elasticity** | 5 | 5 | 4 | 4 | 4 | 5 | 4 | 4 | 3 | 3 | 4 | 5 |
| **Pliability** | 4 | 4 | 4 | 5 | 4 | 4 | 2 | 2 | 3 | 4 | 3 | 3 |
| **Tear resistance** | 4 | 4 | 5 | 5 | 5 | 5 | 5 | 5 | 5 | 5 | 5 | 5 |
| **Needle penetration resistance** | 4 | 3 | 4 | 4 | 5 | 5 | 5 | 4 | 5 | 5 | 5 | 5 |
| **Total Score** | 17 | 16 | 17 | 18 | 18 | 19 | 16 | 15 | 16 | 17 | 17 | 18 |

**Table B**. **Immunohistochemical results of decellularization of reticular and papillary dermis obtained using DMEM.**

| **Staining** | **RETICULAR DERMIS** | | | | **PAPILLARY DERMIS** | | | |
| --- | --- | --- | --- | --- | --- | --- | --- | --- |
|  | Fresh tissue | T0 | T1 | T5 | Fresh tissue | T0 | T1 | T5 |
| Laminin | +++ | +++ | ++ | +  focal | +++ | +++ | ++  diffuse | ++  focal |
| Collagen IV | +++ | +++ | +++ | +++ | +++ | +++ | +++ | +++ |
| CD31 and CD34 | +++  diffuse | +++  diffuse | +++ diffuse | + focal | +++ diffuse | +++ diffuse | +++  diffuse | ++  diffuse |
| Vimentin | +++  diffuse | +++  diffuse | ++ focal | - | +++ diffuse | +++ diffuse | ++  focal | - |
| CD45/CLA | +++ | +++ | +  focal | - | +++ diffuse | +++ diffuse | ++  diffuse | +  diffuse |
| CD68 | +++ | +++ | ++  focal | - | +++ diffuse | +++ diffuse | ++  focal | +  focal |
